# Catalyzing Protein Folding by Chaperones

**DOI:** 10.3390/biology14101450

**Published:** 2025-10-20

**Authors:** Zijue Huang, Scott Horowitz

**Affiliations:** 1 Department of Biochemistry, University of Colorado Boulder, Boulder, CO 80309, USA; 2 Department of Chemistry and Biochemistry, University of Denver, Denver, CO 80208, USA; 3 Knoebel Institute for Healthy Aging, University of Denver, Denver, CO 80208, USA

**Keywords:** protein folding, chaperone, isomerase, quadruplex, RNA

## Abstract

Protein folding is a fundamental process essential for cellular growth and health, yet it is also susceptible to errors that can result in misfolding and disease. This literature review explores the current knowledge of the roles of different factors on protein folding in the cell. We examine the cellular proteostasis network, with a focus on the catalytic actions of prolyl isomerases and molecular chaperones (including RNA G-quadruplexes), which collaborate to guide newly synthesized polypeptides toward their native structures and prevent aggregation. By integrating structural and biochemical insights, this review highlights the current understanding and ongoing questions regarding how chaperones can improve folding times of proteins to physiological pertinent rates.

## Simple Summary

The thousands of different proteins in our cells must fold into intricate three-dimensional shapes to function properly. Aiding proteins to achieve this correct folded state and maintaining that shape over time is a major challenge to the cell, and failure to do so can have disastrous consequences including many diseases. This review article offers background and up-to-date information on how cells aid proteins to achieve their correctly folded states on timescales that allow life to proceed.

## 1. Protein Folding and Problems That Can Occur

Proteins are essential building blocks in the cells, and their proper folding is critical for maintaining a healthy cell environment [1]. As polypeptides emerge from the ribosome, they are unfolded or minimally structured. To become functionally active, these polypeptides must fold into specific three-dimensional conformations. However, protein folding is not always spontaneous or efficient, due to several factors. Typically, folding often involves one or more partially folded intermediates that are unstable and can result in off-pathway events, such as the formation of amorphous aggregates and amyloid fibrils, and targeting by the cellular degradation machinery [2,3,4]. Such challenges stem from the rugged nature of the folding free-energy landscape. This ruggedness is often depicted as a folding funnel, in which protein folding travels down the funnel toward the native, or thermodynamically stable folded state, of the protein at the bottom of the funnel, but many obstacles in energy can populate the funnel. Local minima in energy in the funnel depict intermediates that can trap the protein and prevent its progression to the native state [5].

One of the major challenges of protein folding in the cellular environment is the molecular crowding, where high concentrations of macromolecules increases the risk of protein aggregation [6]. The consequences of problems with protein folding can be severe and often disease related. For example, Alzheimer’s disease and Parkinson’s disease, among many others, are progressive neurodegenerative disordered characterized by the accumulation of misfolded proteins, tau and α-synuclein, respectively.

## 2. Cellular Systems to Aid Protein Folding

The term proteostasis refers to the network of cellular processes that maintain a healthy proteome folding balance [2]. Central to this system is the proper folding of newly synthesized proteins. While many individual folding and degradation mechanisms are well characterized, how these components cooperate in the crowded cellular environment remains poorly understood. Ultimately, a protein’s fate once synthesized within cells follows one of three paths: it folds into its functional state, is targeted for degradation, or misfolds and aggregates. Because misfolding and aggregation can be detrimental, cells have evolved multiple catalytic systems that actively regulate folding at different stages of the process. Among proteins that aid in folding, they can be typically broken into two groups: prolyl isomerases and molecular chaperones. The slow interconversion between cis and trans configurations of proline can delay folding. Peptidyl prolyl isomerases (PPIases) address this issue by catalyzing the conformational switch, allowing proteins to proceed efficiently toward their native state and to modulate protein function.

Complementing this local catalytic action, at a broader scale, many molecular chaperones such as GroEL-GroES, Hsp70, and Hsp90 use ATP to actively promote correct folding, further advanced the concept that chaperones can reshape folding energy landscapes to favor productive folding pathways. Together, PPIases and molecular chaperones play vital roles of the cellular folding machinery to maintain proteostasis. In the following section, we will focus on these mechanisms in detail.

## 3. Catalysis by Prolyl Isomerases

Proline is one of the 20 standard amino acids, but has unique backbone configuration properties among amino acids. More than 99% of the peptide bonds between amino acid residues other than proline are in the trans configuration. While most prolines also favor the trans configuration, the cis configuration is often essential for a protein to adopt its native structure [7,8]. This cis-trans isomerization involves rotation around a partial double bond (O=C-N) involving the imino nitrogen, and is energetically demanding with a high activation enthalpy, making the process inherently slow. A critical folding barrier arises in partially folded intermediates, where prolines adopt incorrect isomers that often persist and block progression to the native state. Garel and Baldwin first demonstrated this in studies on ribonuclease A (RNase A), showing that both fast and slow refolding pathways, when disulfide bonds remain intact, contribute to the recovery of fully active RNase A [9]. Later, the crystal structure of RNase A revealed that, in its folded state, two prolines are in cis conformation (Pro93 and Pro114), while two others in trans conformation (Pro41 and Pro117) [10]. This structural insight was consistent with previous studies showing the role of proline isomerization in the folding process. Specially, it suggests that non-native prolyl isomers do not interfere with the early stages of refolding, and that cis/trans prolyl isomerization is not the initial step in the folding process [11,12]. Instead, it often represents a rate-limiting step in the protein folding pathway [13,14,15].

Importantly, this regulatory role of proline isomerization extends beyond folding substrates to modulate chaperone function itself. For example, Hsp70 is proposed to be allosterically regulated by isomerization at Pro143, a residue in the ATPase domain critical for stabilizing the open conformation of the substrate-binding pocket [16,17,18]. Hsp70 exists in two stable conformations: an ATP-bound open state and an ADP-bound closed state. Substituting Pro143 increases the enthalpy of ATP hydrolysis and destabilizes this conformational switch, leading to a faster and less controlled transition between the ATP- and the ADP-bound form [18]. In addition to their enzymatic activity, PPIases including immunophilins such as cyclophilins and FK506 binding proteins (FKBPs) also act as co-chaperones. They interact with Hsp90 through a tetratricopeptide repeat (TPR) motif binding its C-terminal sequence, an essential interaction required for steroid hormone receptor complexes [19,20,21].

Altogether, PPIases catalyze slow proline isomerization steps (rate-limiting steps) and partner with major chaperone systems. Their presence in both the cytosol and periplasm, indicating their importance in maintaining homeostasis across diverse cellular environments.

## 4. Overview of Molecular Chaperones

Beyond enzymatic catalysis of bond rearrangements, cells also rely on ATP-driven folding machines called molecular chaperones, which catalyze protein folding on a global scale. These proteins are often categorized by molecular weight and are commonly referred to as heat-shock proteins due to their upregulation under stress conditions such as heat shock or oxidative stress. Molecular chaperones assist in guiding nascent protein toward their correctly folded, functional states through a variety of proposed mechanisms (Table 1). Chaperones are classified into two categories: ATP-dependent chaperones, often termed foldases, use ATP to actively promote protein folding. ATP-independent chaperones, often termed holdases, prevent off-pathway misfolding and aggregation by stabilizing unfolded or partially folded proteins.

Among ATP-independent chaperones, small heat-shock proteins (sHsps) represent a key class of holdases and serve as a first line defense against protein misfolding [4]. Rather than actively refolding protein substrates, sHsps bind and stabilize misfolded proteins to prevent aggregation. They typically exist as large inactive oligomers, but under stress conditions, they shift toward smaller species, often dimers, which are more competent for substrate binding [22]. However, the release and subsequent refolding of protein substrates bound by sHsps does not occur spontaneously and instead requires the cooperation of ATP-dependent chaperones.

One such system is the ATP-regulated Hsp70 (for example, bacterial DnaK), which interacts with newly synthesized polypeptides. Hsp70 functions with co-chaperones of Hsp40 (for example, bacterial DnaJ) and nucleotide exchange factors (for example, bacterial GrpE). In its ATP-bound open state, Hsp70 binds unfolded or partially folded protein substrates through linearized polypeptide stretches [23]. ATP hydrolysis is accelerated by Hsp40, induces a conformational change to a closed, ADP-bound state that clamps the substrate [2,24]. Subsequently, nucleotide exchange factors promote the exchange of ADP-ATP, reopening the substrate binding domain and releasing the folding intermediates [25]. Proteins that fail to fold efficiently through Hsp70-associated cycling are often handed off to chaperonins which provide encapsulated environment to support productive folding. While Hsp70 acts early in the folding process, the chaperonins are suggested to assist in the final folding of protein substrates that failed to reach native state with Hsp70 alone [26,27].

In the cell, it is specifically likely that the mechanism of Hsp70 is closely linked to that of Hsp90. Like Hsp70, Hsp90 cycles ATP to drive large conformational changes, but for many years it was unclear what the purpose of Hsp90’s ATP cycles was, as the steps of its chaperone cycle were not entirely tied to its nucleotide binding state like in the case of Hsp70 [28]. It was often thought that Hsp90 acts as a late-stage folding enhancer, but recent research has offered more specificity to the mechanism with findings suggesting that a major part of Hsp90’s duties are to interface with Hsp70 and provide a release for refolding, with major involvement by Hsp90 co-chaperones to aid the process [29,30,31]. In particular, the bridging co-chaperone Hop (Hsp70/Hsp90-organizing protein, also known as stress-inducible protein 1 in yeast) and p23 modulate the dynamics between inactive glucocorticoid receptor (GR) “loading” complex bound to Hsp70/Hsp90/Hop and active GR “maturation” complex associated with Hsp90/p23. Therefore, it is possible that Hsp70 and Hsp90’s functions are highly co-dependent.

The chaperonin system GroEL-GroES has been the subject of rigorous investigation through both theoretical and experimental approaches [24,32,33,34,35,36,37,38]. Its significance lies not only in its broad substrates, serving an approximately 10% of newly synthesized cytosolic proteins [39], but until recently, was also the only well-characterized case where true catalytic acceleration of protein folding had been demonstrated [40].

## 5. Catalysis by GroEL-GroES (Chaperonin)

The bacterial GroEL has two rings, cis and trans, each composed of seven identical subunits. These large oligomeric assemblies form nanocage-like structures that isolate substrate proteins during refolding. The overall reaction cycle of GroEL-GroES starts when non-native substrate protein binds to the trans ring of GroEL, followed by ATP-dependent GroES binding, which encapsulates the substrate protein within the cavity. Inside the cavity, the protein is free to fold in a protected environment for the duration ATP hydrolysis, typically involving hydrolyzing 7 ATP molecules in the cis ring. Upon ATP hydrolysis, ADP and GroES release, and the folded protein exits the cavity, completing the cycle [41]. In eukaryotes, the Chaperonin-Containing TCP-1 (CCT), also known as TCP-1 ring complex (TRiC), utilizes a similar process to fold proteins [42]. Both types of chaperonins form double ring structures that provide an isolated environment for proteins to fold correctly.

However, key questions remain whether GroEL provides passive protection or actively catalyzes folding steps, and under what circumstances it accelerates or alter folding pathways [32,35,43]. Here, we review several proposed mechanisms of GroEL function, both catalytic and noncatalytic models. These models include steric confinement to prevent aggregation, active unfolding of misfolded conformations, direct promotion of folding and enhancement of polypeptide collapse.

### 5.1. Passive Anfinsen Cage/Folding Indirectly

Christian Anfinsen in 1973 demonstrated that, for at least one protein, that the amino acid sequence contained all of the information needed to encode the tertiary structure of the protein. Furthermore, the protein could fold spontaneously to this state when given conditions that allow refolding [44]. After the discovery of GroEL, Horwich et al. coined the term “Anfinsen cage” to describe a model in which the nanocage does not directly catalyze folding but instead passively prevents misfolding, providing a protected, aggregation-free environment that allows proteins to fold spontaneously [45]. While this concept appears to clash with the concept of a foldase, which actively accelerates folding through ATP hydrolysis, in this model GroEL is suspected to use ATP purely to drive full encapsulation and isolation for folding. Horwich et al. proposed that the GroEL-GroES complex acts as a passive anti-aggregation device, primarily through the non-stick inner wall of the cis ring, promoting folding indirectly by sequestering non-native proteins in an enclosed cavity. Isolating the substrate in a confined space, free from the risk of intermolecular aggregation, allows folding to proceed without interference [44,46,47,48].

### 5.2. Reversing Misfolding/Iterative Annealing

GroEL-GroES has also been proposed to unfold misfolded protein conformation or kinetically trapped intermediates, thereby providing substrates with another opportunity to refold correctly [49,50,51,52]. This ‘iterative annealing’ model suggests that chaperones repeatedly bind kinetically trapped client proteins, disrupt their incorrect structures randomly and release them, allowing multiple chances to find the path to the most thermodynamically stable state [53]. Lin et al. used Rubisco as their model and found that GroEL unfolds it in two phases: an initial slow passive unfolding of Rubisco upon capture by the trans ring of GroEL, followed by a rapid ATP-driven forced unfolding [51]. This sequential unfolding process enhances the productive refolding of Rubisco. In some cases, large substrates that cannot fit inside the GroEL cavity bind to the trans ring and are directly ejected into free solution [54,55,56]. Thus, trans-only GroEL ring, which cannot encapsulate protein substrate, still enhances the folding of Rubisco and another common GroEL client, Malate Dehydrogenase [55]. This effect is explained by partial unfolding of these proteins on the open trans ring prior to their ejection. These findings provide direct experimental evidence that GroEL-mediated unfolding is functionally coupled to the enhancement of protein folding.

### 5.3. Accelerating Folding Directly

Earlier studies showed GroEL-GroES’s ability to enhance folding rates across diverse protein substrates. For example, Brinker et al. demonstrated that GroEL-GroES enhances the folding rate for bacterial Rubisco [57]. Additionally, Tang et al. reported a ten-fold acceleration in folding rate for a double mutant of maltose-binding protein (dmMBP) [41]. Chakraborty et al. later proposed that this effect may stem from steric confinement within the closed GroEL cavity, which reduces conformational entropy therefore effectively lowering the folding barrier [41,57,58]. A particularly striking study comes from Libich et al. [40] in 2015 that provided the first direct evidence of catalytic acceleration of folding within the GroEL cavity. Using thermodynamically unstable variants of the Fyn SH3 domain as a model substrate, they employed comprehensive NMR analyses and showed that the transition from SH3 folding intermediate to its native state occurred while the protein was still encapsulated by GroEL. Remarkably, this transition was catalyzed, resulting in a ten-fold increase in folding rate [40]. While Libich et al. pointed out the catalytic potential of GroEL cavity, most recent work has expanded our understanding of GroEL on folding dynamics. In a study by Naqvi et al., optical tweezers methods demonstrated that the chaperonin system promotes maltose-binding proteins (MBPs) folding transitions by enhancing polypeptide collapse [59]. Notably, this effect may not depend on the closed cavity characteristic of GroEL.

Whether all four of these mechanisms are in fact relevant in cells is still unclear, as varying experimental conditions and diverse client proteins likely affect the mechanism observed. GroEL may employ different strategies depending on the condition, reflecting its role in maintaining proteostasis.

Taken together, molecular chaperones are essential modulators of protein homeostasis. The chaperonins could help overcome both entropic and enthalpic barriers within a rugged folding landscape. Traditionally, ATP-dependent chaperones use ATP hydrolysis to drive large conformation changes that facilitate protein folding, while ATP-independent chaperones act by holding aggregation-prone proteins and later transferring them to ATP-dependent chaperones for active folding. However, there exists another scenario in which molecular chaperones remain continuously bound to their client proteins in a more passive manner during the folding process. In the next section, we will examine these folding while bound mechanisms in greater detail.

## 6. Folding While Bound: SecB and Spy

While we have discussed how the GroEL cage catalyzes folding by accelerating specific steps, emerging evidence suggests that not all folding is catalyzed. Binding to a chaperone does not universally promote faster folding. For example, as described above, GroEL can accelerate folding for Fyn SH3 domain. However, in other cases, including studies on barnase, the bound state to GroEL actually slows down folding [60]. This variability highlights a broader theme: folding while bound has typically been observed to slow down folding, with the amount of slowdown depending on the system. While ATP-dependent GroEL show both outcomes, ATP-independent chaperones have traditionally been viewed as passive holdases. In addition to GroEL, folding while bound has been observed with other chaperones such as SecB and Spy.

*E. coli* SecB functions both in membrane translocation and as a cytosolic chaperone [61,62,63]. As seen with GroEL and barnase, SecB also slows the folding of barnase while it remains tightly bound, with nanomolar affinity [64,65]. However, unlike GroEL, SecB does not undergo large conformational changes for substrate binding and release. Subsequent studies that used a different protein substrate, matured MBP, showed that it can also folding while bound to SecB. In contrast, the pre-matured MBP does not. Notably, the binding affinity of SecB for matured MBP is weaker (80 nM) compared to that for the premature form (0.8 nM), suggesting that folding while bound is influenced by both the substrate and binding strength [66].

Spy (spheroplast protein Y) is a small periplasmic chaperone from *E. coli*. Spy’s structure is a small cradle with a flexible surface, and due to its small size, Spy has been used as a model for studying how molecular chaperones help proteins fold [67]. Spy forms a positively charged surface and a large hydrophobic surface in the middle of the cradle [68]. The chaperone activity of Spy has been elucidated using protein folding model Im7 in great detail. Im7 folds via a three-state model that includes an on-path intermediate [69,70,71,72,73,74]. Spy was able to bind all three states of Im7 with affinity in the micromolar range and Im7 folded while continuously bound to Spy [72]. Of note, like SecB and GroEL, Spy did slightly slow down Im7 folding. Koldeway et al. showed that Spy initially engages unfolded Im7 through rapid, long-range electrostatic attraction. This is followed by stabilization of the Spy-Im7 complex via hydrophobic contacts. The primary driving force for Im7 folding is hydrophobic collapse. Once the hydrophobic residues of Im7 begin to bury into its core, Spy-Im7 affinity decreases ultimately leading to the release of the folded Im7. This interpretation was consistent with structural investigation of Im7’s folding while bound to Spy via both NMR and dynamic crystallography [70,75].

The folding-while-bound observations with Im7 left it unclear whether this mechanism was general. To address this, Wu et al. later used a different target protein, the β-sheet-rich Fyn SH3, which unlike the helical Im7, follows a two-state folding mechanism [76]. Despite the structural and mechanistic differences, Fyn SH3 was also shown fold while bound to Spy, remaining loosely but continuously in contact with the surface of Spy. Notably, under conditions of excessively strong Spy-SH3 binding, the folding rate decreases, suggesting that productive folding is dependent on weak interactions.

Unlike most protein chaperones in the cytosol, Spy functions without the driving force of ATP. This raises a potential issue: without an energy driven mechanism, Spy could bind too tightly (high affinity) to unfolded proteins and fail to release them, therefore shifting the folding equilibrium to the unfolded direction, and turning Spy into a holdase [77]. In general, the effectiveness of Spy as a chaperone is substrate specific, its binding requires being strong enough to prevent aggregation, as seen with apoflavodoxin (0.35 μM), yet weak enough to allow productive folding and timely release, as demonstrated with Im7 and SH3 (4.7 μM and 2.9 μM, respectively). This highlights the critical role of binding affinity in modulating folding kinetics for ATP-independent chaperones like Spy.

## 7. Chaperone RNAs

Protein folding research in recent decades has largely focused on protein-based chaperones. However, recent studies have expanded this view, demonstrating that other macromolecules, such as nucleic acids and polyphosphate, also possess chaperone activity [78,79,80]. Docter et al. showed nucleotide homopolymers have a range of holdase activity. Among them, polyU was particularly effective, suppressing protein aggregation up to 300-fold more efficiently than GroEL by weight. Furthermore, polyU was shown to promote protein folding in a passive manner prior by collaborating with Hsp70, suggesting a co-chaperone role in the folding pathway [79].

Their findings opened the door to the idea that nucleic acids can contribute more broadly to cells. Supporting this, it has been shown that approximately 10% of the proteome relies on RNA to maintain solubility, even among proteins not classified as RNA-binding [81]. Further investigation is needed to uncover the full extent of nucleic acid functional diversity.

### 7.1. G-Quadruplexes (G4s) as Chaperones

**Figure 1:**
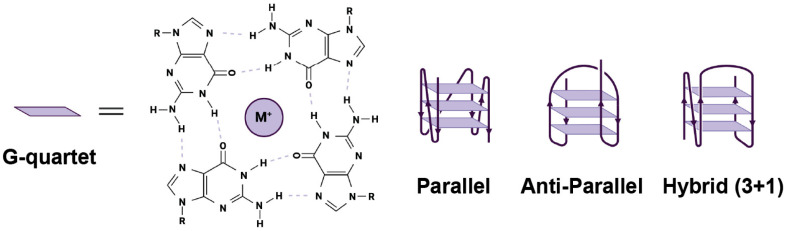
Topologies of G4s. G-quadruplexes are four-stranded nucleic acid structures formed by guanine-rich sequences and stabilized by a central metal, often potassium. Depending on strand orientation, diverse topologies can form, include parallel, anti-parallel, and hybrid conformations.

Building on these insights, recent work from our group and others have shown that G4s are powerful modulators of protein folding, oligomerization and aggregation [82,83,84,85]. G4s are four-stranded secondary structures formed by guanine-rich in both DNA and RNA. They are characterized by the formation of stacked G-quartets, which are planar arrangements of four guanines stabilized by Hoogsteen hydrogen bonding and monovalent cations at the center such as potassium and sodium. Structurally, G4s are diverse and can adopt multiple topologies depending on strand orientation. These include the parallel conformation, with all four strands in the same direction, the anti-parallel conformation, with alternating strand directions, and the 3+1 hybrid conformation, with three strands in the same direction and one in the opposite direction (Figure 1). G4s have previously been characterized as regulators of transcription, translation, and splicing [86]. Although the full extent of G4 formation in cells remains unclear, G4s have been identified within the rRNA extensions of ribosomes, suggesting a high local concentration of these structures at the original folding site [87].

To test the hypothesis that G4s function as chaperones, specifically in a holdase capacity, Begeman et al. screened over 300 randomized single-stranded DNAs using a thermal aggregation assay [83]. This in vitro screen assessed each sequence’s ability to prevent aggregation under heat stress. Remarkably, G4-forming sequences were significantly more potent than non-G4 sequences, showing an order of magnitude greater efficiency in suppressing aggregation. G4s also demonstrated broad holdase activity across a range of protein substrates, including citrate synthase (CS), luciferase (Luc), L-malate dehydrogenase (MDH), and L-lactate dehydrogenase (LDH), indicating that their role is general rather than substrate specific. Litberg et al. later focused on how G4s influence protein aggregation, primarily examining G4 sequences LTR-III and PARP1 and their chaperone activity [88,89]. Their findings implicated overall structural topology, particularly the 3+1 hybrid conformations, as important in suppressing aggregation. Notably, when potassium ions were replaced with lithium, which does not support G4 formation, holdase activity was abolished, highlighting the critical importance of the G4 structure itself. They also observed a trend in chaperone activity that correlated with NMR chemical shift changes in residues located directly above the G4 plane, suggesting that the accessibility of the plane plays a critical role. These finding point to the importance of G4 core itself and highlight that direct contacts between the G4 and adjacent regions maybe key to its function [90,91,92].

These G4s also have an interesting property that they form larger self-oligomers, and oligomerization appears to play an intriguing role in chaperone function [83]. Not all of the G4s were disrupted by substitution with lithium, and in some cases, the lithium buffer condition led only to the loss of higher order G4 oligomers. Interestingly, these cases still lost considerable chaperone activity, suggesting the importance of higher-order structures in the chaperone activity. This observation is consistent with previous reports that the chaperone-like functions of bulk nucleic acids rely on oligomerization [85]. Such oligomerization could act as a kinetic trap to prevent further aggregation or, in some cases, accelerate it. Notably, the oligomerization of G4s is similar to the behavior of mechanism observed in small heat shock proteins [4].

### 7.2. G4s Directly Impacting Protein Folding

As mentioned above, polyU RNA had been shown to aid Hsp70’s folding cycle passively, but was unable to directly improve protein folding on its own [79]. But could G4s do so? In parallel, to investigate how G4s assist protein folding, we selected TagRFP675, a red fluorescent protein, as a folding biosensor [93]. Fluorescent proteins are useful in this context because their fluorescence is dependent on the correct folding of their β-barrel structure and the proper maturation of the chromophore [94,95]. Like wild-type GFP, many red fluorescent proteins, including TagRFP675, fold inefficiently in *E. coli* under standard growth conditions. The inability of TagRFP675 to fold on its own in *E. coli* is consistent with previous literature on the folding challenges of fluorescent proteins [96,97,98,99,100].

Unlike wild-type GFP, TagRFP675 possesses a slight positive net charge, which allows it to interact with nucleic acids including G4s. This feature made TagRFP675 especially well-suited for probing the effect of nucleic acid-based chaperones on protein folding. We took advantage of this property by co-expressing TagRFP675 with either G4s or known protein chaperones in *E. coli*. Proper folding of TagRFP675 leads to fluorescence at its native excitation/emission wavelength 598/675 nm, providing a quantitative readout of folding efficiency in the cellular environment. Co-expression with either known chaperones or G4s significantly enhanced proper protein folding, providing in vivo evidence that G4s could contribute to a favorable folding environment in cells [82,83].

Although G4s could act as holdases and aid the protein folding environment, it was unclear whether G4s could directly enhance folding in vitro or in cells. Using protein refolding experiments, Son et al. found that RNA and DNA G4s can rescue kinetically trapped intermediates of TagRFP675 in vitro and help its transition to the native state. In cells, where degradation and translation were paused, the addition of RNA G4s improved protein folding [82], indicating that G4s directly promote folding. These findings led to the creation of a model for how G4s could act as chaperones. In eukaryotes, RNA G4s prefer to form under stress conditions [101], when proteins are also prone to unfolding, suggesting RNA G4s as molecular chaperones that aid protein folding during stress and release them when stress resolves.

How did the G4s aid protein folding? It was unclear if proteins fold while bound to RNA G4s, and if so, how the rates of folding were affected. Therefore, we set out to determine the mechanism of a protein folding in the presence of a G4. First, we had to characterize the folding of the test protein TagRFP675, in considerably greater detail. Using protein refolding and unfolding experiments with global data modeling, we determined the folding mechanism of TagRFP675. The mechanism showed two intermediates: an off-pathway intermediate I_2_, which interconverts with both the unfolded state (U) and the on-path intermediate (I_1_). We then added the G4 and examined the change in mechanism. With the G4, the net folding rate constant was approximately 3-fold higher and no I_2_ was observed, keeping the protein on-pathway for folding. Interestingly, all of the folding and unfolding steps while bound to the G4 were higher than that of the protein folding alone, thereby qualifying the G4 as a protein folding catalyst. Furthermore, because of the high thermal stability of the G4, we had the opportunity to measure the temperature dependence of folding while bound to the G4 and estimate the changes in the driving forces of folding. The acceleration of the U to I_1_ transition by the G4 was primarily driven by a change in heat capacity, whereas the acceleration of the I_1_ to N transition was instead driven by a reduction in the entropic barrier [102].

Despite these insights, key questions remain about how G4s work as chaperones in protein folding. For example, what is the molecular mechanism by which G4s interact with the target protein? And what role does G4 oligomerization play in modulating this interaction? Our findings have suggested that oligomerization appears to be a critical factor influencing the chaperone activity of G4s, but speculation is currently required as to the reason. One possibility is that G4 oligomers may form a pseudo-cage structure reminiscent of the GroEL folding chamber, creating a similar confined environment for the client protein. This cage-like structure could shield partially folded intermediates and promote active folding. Another possibility is that oligomerization introduces significant structural diversity into the G4 population. This conformational heterogeneity increases the number of accessible conformational microstates and presents more binding sites for the client protein. As a result, binding to such a dynamic and heterogeneous ensemble of G4s would be entropically favored compared to interacting with a single, rigid G4 structure. This higher configurational entropy may enhance the ability of G4 to engage more effectively with unfolded or partially folded proteins. Finally, although we observed a strong correlation between G4 oligomerization and chaperone activity, this relationship does not necessarily reflect direct causality. It remains possible that both oligomerization and chaperone function are driven by shared underlying structural features or are modulated by other external factors such as ionic strength and environmental condition.

## 8. Areas of Future Questions

There are multiple areas of interest to continue exploring in relation to folding while bound to RNA. Prior studies have reported that stable G4s structures can form in human rRNA [87,103]. Given the high local concentration of G4s within the ribosome and combined with our in vitro data showing that G4s can catalyze protein folding, this raises the intriguing possibility that the original site ribosome itself could serve as a natural platform for G4 oligomerization. If oligomerization is indeed the key to G4-assisted folding, then the dense clustering of G4s in ribosomal RNA could facilitate the assembly of functional G4 oligomers, thereby enhancing co-translational folding in vivo. Thus, G4s may not only serve as small regulators within the ribosome, but G4s may also actively contribute to shape the folding landscape and to protein homeostasis during translation especially under stress condition when protein folding is most vulnerable.

How might the folding while bound to G4 phenomenon affect misfolding disease? G4s have also been observed to accelerate protein aggregation [104,105,106]. This dual behavior also raises an important question on the broader level: how general is this balancing effect between folding and aggregation control? Under chronic stress where G4s are not unfolded, they accumulate in the brain and their oligomerization activity could become detrimental [106]. Elucidating how G4s could shift between protective and pathological in protein folding remains an important area of future investigation.

By exploring these topics, we move closer to understanding how short nucleic acids, both DNA and RNA, can serve as dynamic modulators of protein behavior in vitro and in cells. Ultimately, these future directions not only deepen our mechanistic understanding but also open new avenues for targeting protein misfolding in neurodegenerative diseases.

## 9. Conclusions

Although the exact nature of how catalysis of protein folding occurs is still controversial in at least the case of GroEL, the existence of multiple different macromolecules that can catalyze protein folding suggests that it is a relatively commonly used biological mechanism. Many questions still remain to be answered, and it is possible that catalysis of protein folding still may be used by cells considerably more than previously suspected.

## Figures and Tables

**Table 1:** Currently proposed mechanisms of molecular chaperones.

Mechanism	GroEL	TRiC	Hsp70	Spy	SecB
Anfinsen Cage	x	x			
Iterative Unfolding with ATP	x	x	x		
Accelerate Folding	x	x			
Folding While Bound	x			x	x

## Data Availability

No new data were created or analyzed in this study. Data sharing is not applicable.
